# Kinetics and Mechanism of Liquid-State Polymerization of 2,4-Hexadiyne-1,6-diyl *bis*-(*p*-toluenesulfonate) as Studied by Thermal Analysis

**DOI:** 10.3390/polym16010007

**Published:** 2023-12-19

**Authors:** Andrey Galukhin, Alexander Kachmarzhik, Alexander Rodionov, Georgy Mamin, Marat Gafurov, Sergey Vyazovkin

**Affiliations:** 1 Alexander Butlerov Institute of Chemistry, Kazan Federal University, 18 Kremlevskaya Street, 420008 Kazan, Russia; sasha.kachma@mail.ru; 2 Institute of Physics, Kazan Federal University, 18 Kremlevskaya Street, 420008 Kazan, Russia; rodionovshurik@yandex.ru (A.R.); georgemamin@gmail.com (G.M.); mgafurov@gmail.com (M.G.); 3 Department of Chemistry, University of Alabama at Birmingham, 901 S. 14th Street, Birmingham, AL 35294, USA

**Keywords:** diacetylenes, polymerization, azide–alkyne cycloaddition, isoconversional analysis, differential scanning calorimetry, electron paramagnetic resonance

## Abstract

A detailed investigation of the liquid-state polymerization of diacetylenes by calorimetric (DSC) and spectroscopic (in situ EPR) thermal analysis techniques is performed. Isoconversional kinetic analysis of the calorimetric data reveals that liquid-state polymerization is governed by a well-defined rate-limiting step as evidenced by a nearly constant isoconversional activation energy. By comparison, solid-state polymerization demonstrates isoconversional activation energy that varies widely, signifying multistep kinetics behavior. Unlike the solid-state reaction that demonstrates an autocatalytic behavior, liquid-state polymerization follows a rather unusual zero-order reaction model as established by both DSC and EPR data. Both techniques have also determined strikingly similar Arrhenius parameters for liquid-state polymerization. Relative to the solid-state process, liquid-state polymerization results in quantitative elimination of the *p*-toluenesulfonate group and the formation of *p*-toluenesulfonic acid and a polymeric product of markedly different chemical and phase composition.

## 1. Introduction

**Figure 1:**
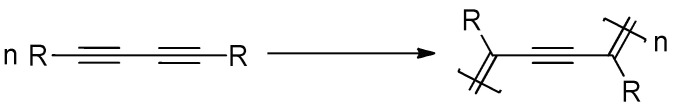
Scheme of the solid-state polymerization of diacetylenes to polydiacetylenes.

Polydiacetylenes are extensively studied because of their unique structural, spectral, and optical properties [1]. Their highly conjugated structure being exposed to chemical [2,3,4], thermal [5,6,7], or mechanical [8,9,10] stimulus undergoes conformational changes, which alter chromatic properties of the material. Solid-state polymerization of diacetylenes is a topochemical reaction that requires specific orientation of the monomer molecules in the crystalline phase [11]. It proceeds as a 1,4-addition with intermediate formation of various radical species [12] to yield a rigid polymer backbone containing conjugated enyne fragments (Figure 1). Polymerization is usually initiated by raising temperature or supplying UV radiation. According to the literature data, solid-state thermally stimulated polymerization of diacetylenes demonstrates pronounced autocatalytic behavior [13,14,15]. When investigated in isothermal conditions, it goes through an induction period followed by a rapid reaction. In the solid-state, the reported ratio of the maximum reaction rate to the reaction rate during the induction period ($r_{max}/r_{i}$), used as a characteristic of autocatalytic processes, reaches ~100 according to calorimetric [16] and spectroscopic [17] studies.

For thermally induced liquid-state polymerization of diacetylenes, the reported results seem to differ markedly from those related to the solid-state reaction. It was found that for a set of diacetylenes subjected to the liquid-crystalline-state polymerization, the heat of polymerization exceeds dramatically that of the reaction in the crystalline phase (~240 vs. ~140 kJ mol^−1^) [18]. A similar value of polymerization heat (~270 kJ mol^−1^) was reported for liquid-state reaction of some rigid diacetylenes [19]. It was also reported [19] that the induction period is absent in the case of the liquid-crystalline-state polymerization and that the properties of the polymers obtained differ from those for the materials obtained in the melt. It was tentatively suggested [19] that the structures of the polymers obtained in liquid-crystalline and isotropic melt polymerization differ as well. On the other hand, later studies of the molten state polymerization of unsymmetrically substituted diacetylenes by FTIR concluded that both reactions proceed in the same manner [20].

Such intriguing differences between the polymerization processes in ordered and disordered phases and the absence of general insights into this issue have motivated us to undertake a detailed study of the liquid-state polymerization of diacetylenes. As the subject of our work, we have chosen 2,4-hexadiyne-1,6-diyl *bis*-(*p*-toluenesulfonate) because, unlike other diacetylenes, its polymerization kinetics in the solid state are well studied and documented [16,17,21], which makes it a preferable reference system for our comparative liquid-state study. We have used differential scanning calorimetry (DSC) to study the polymerization process. The obtained calorimetric data were then subjected to comprehensive isoconversional kinetic analysis [22] in order to determine the activation energy, pre-exponential factor, activation entropy, and reaction model of the process. The calorimetric data were complemented by in situ EPR measurements. A combination of these two techniques allowed us to gain valuable insights into the mechanism of liquid-state polymerization. 

## 2. Materials and Methods

### 2.1. Materials

**Figure 2:**
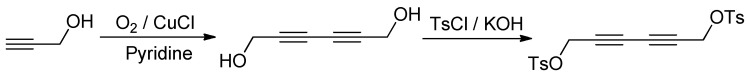
Synthesis of 2,4-hexadiyne-1,6-diyl *bis*-(*p*-toluenesulfonate) from propargyl alcohol via hexa-2,4-diyne-1,6-diol.

Propargyl alcohol (99%, Sigma-Aldrich, Waltham, MA, USA), copper (I) chloride (99%, Sigma-Aldrich, Saint Louis, MI, USA), ammonium chloride (99.5%, Chimmed, Moscow, Russia), pyridine (99.5%, EKOS-1, Moscow, Russia), *p*-toluenesulfonyl chloride (TsCl, 99+%, Acros Organics, Geel, Belgium), potassium hydroxide (86%, Chimmed, Moscow, Russia), sodium sulfate (anhydrous, 99.5%, Chemprom-M, Yaroslavl, Russia), methanol (99.5%, Vekton, Saint Petersburg, Russia), dichloromethane (99.5%, Chemprom-M, Yaroslavl, Russia), benzene (99.8%, EKOS-1, Moscow, Russia), and silica gel (60 Å, Machery-Nagel, Duren, Germany) were used as received. Acetone (99.5%, Chemprom-M, Yaroslavl, Russia) was distilled over phosphorus pentoxide before use. Tetrahydrofuran (99.5%, EKOS-1, Moscow, Russia) and diethyl ether (99%, Kuzbassorgchem, Kemerovo, Russia) were distilled over potassium hydroxide before use. Deionized water (18.2 MΩ) was obtained using an Arium mini-instrument (Sartorius, Gottingen, Germany). Target monomer 2,4-hexadiyne-1,6-diyl *bis*-(*p*-toluenesulfonate) was synthesized according to Figure 2.

Hexa-2,4-diyne-1,6-diol was synthesized from propargyl alcohol with Glaser cross-coupling [23]. Propargyl alcohol (20 g, 0.357 mol), pyridine (7.2 mL, 0.089 mol), copper (I) chloride (0.424 g, 4.28 mmol), and 200 mL of methanol were added to a round-bottom flask equipped with condenser and magnetic stirring bar. Obtained mixture was heated to 35 °C and then oxygen was bubbled through it for 4 h. After that, the reaction mixture was poured into a saturated aqueous ammonium chloride solution and extracted with several portions of diethyl ether. Combined diethyl ether extract was washed with 10 wt % sodium carbonate solution and dried over anhydrous sodium sulfate. After that, diethyl ether was evaporated, and the obtained crude product was recrystallized from benzene. The yield was 27%. The synthesized compound was additionally purified using silica gel column chromatography with diethyl ether as an eluent before the next synthetic step. ^1^H NMR (D_2_O): δ (ppm) 4.28 (4H, s, CH_2_). ^13^C NMR (D_2_O): δ (ppm) 49.83, 68.74, 77.33.

2,4-Hexadiyne-1,6-diyl *bis*-(*p*-toluenesulfonate). The target monomer is sensitive to light; therefore, its synthesis, isolation, and purification procedures were carried out in the dark using a red lamp. Hexa-2,4-diyne-1,6-diol (2.00 g, 18.2 mmol) and *p*-toluenesulfonyl chloride (9.00 g, 47.2 mmol) were dissolved in 60 mL of tetrahydrofuran under continuous stirring, then the solution of potassium hydroxide (3.67 g, 65.3 mmol) in 30 mL of water was added dropwise while cooling the mixture on the water bath. Then, the reaction mixture was stirred for 5 h at room temperature. The reaction mixture was poured into 150 mL of ice water, producing red precipitate. The precipitate was filtered off and washed with ice water. The obtained product (the monomer) was purified using silica gel column chromatography with dichloromethane as an eluent. The yield was 61%. T_m_ (onset) was measured to be 94 ± 2 °C and the heat of melting was 36.3 ± 0.8 kJ mol^−1^. ^1^H NMR (CDCl_3_, δ, ppm): 2.45 (6H, s, CH_3_), 4.73 (4H, s, CH_2_), 7.35–7.80 (8H, m, Ar-H). ^13^C NMR (CDCl_3_, δ, ppm): 21.73, 57.46, 71.98, 72.18, 128.16, 130.01, 132.59, 145.60. IR (cm^−1^): 1361, 1368 (S=O), 1172 (C–O) (Figures S1–S3).

### 2.2. Methods

A Bruker AVANCE III NMR spectrometer (Billerica, MA, USA) was run at 400 MHz to produce the ^1^H and ^13^C NMR spectra using CDCl_3_ as a solvent. The reported chemical shifts, delta (δ), are in units of parts per million (ppm). A Bruker Vertex 70 FTIR spectrometer was used to record IR spectra. 

A heat flux DSC 3+ (Mettler-Toledo, Greifensee, Switzerland) was employed for calorimetric measurements. Temperature, heat flow, and tau-lag calibrations were accomplished by utilizing indium and zinc standards. Nonisothermal experiments were performed under argon flow (80 mL min^−1^) in the temperature range 25–250 °C at the heating rates of 1.0, 2.0, 4.0, and 8.0 °C min^−1^ in 40 µL aluminum pans with pierced lids. Isothermal measurements were performed at 70, 75, 80, and 85 °C with the same DSC instrument in 40 µL aluminum pans with pierced lids. The mass of the sample in each run was 5.0 ± 0.1 mg. The mass loss of the sample during polymerization was evaluated by simultaneous TGA-DSC measurement with Netzsch STA 449 F1 Jupiter thermal analyzer (Selb, Germany). The measurement was conducted at 10.0 °C min^−1^ under argon flow of 75 mL min^−1^, as recommended by the manufacturer. The mass of the sample was ~5 mg. The mass loss of the sample in the temperature range of 50–200 °C did not exceed 2.5% (Figure S8).

A MiniFlex 600 diffractometer (Rigaku, Tokyo, Japan) with a D/teX Ultra detectorX-ray was used for powder diffraction (XRPD) measurements. The instrument radiation source was Cu Kα1 operated at 40 kV, 15 mA. XRPD data were collected at ambient temperature at the diffraction angle 2θ varying from 2° to 100° at 0.02° steps and 0.24 s exposure time at each point without sample rotation.

In situ EPR measurements were performed with microwave X-band (9.61 GHz) Bruker ESP–300 spectrometer equipped with a Bruker ER 4111 VT temperature attachment in a continuous wave mode. Monomer samples were placed in glass ampoules hermetically sealed in the atmosphere of argon. The concentration of paramagnetic centers in the final samples was estimated in a double resonator ER 4105DR (Bruker) using a standard 2,2-diphenyl-1-picrylhydrazyl calibration sample.

## 3. Calculations

Kinetic analysis was carried out according to the guidelines of the ICTAC Kinetic Committee [22]. The flexible integral isoconversional method of Vyazovkin [24] was employed to evaluate the activation energy *E_α_* as a function of conversion (*α*) for the liquid-state polymerization studied in nonisothermal conditions. The use of this method eliminates the systematic error in *E_α_* found for rigid integral methods when *E_α_* reveals a significant dependence on *α* [24]. The advantage arises from the flexible integration conducted over small intervals of Δα, within which the variability of *E_α_* can be neglected. For the present calculations, *Δα* was set as small as 0.01. Within each Δ*α*, *E_α_* was determined as the value that corresponds to the minimum of the following function:

(1) $$\Psi \left(E_{\alpha }\right)=\sum_{i=1}^{p}\sum_{j\neq i}^{p}\frac{J\left[E_{\alpha },T_{i}\left(t_{\alpha }\right)\right]}{J\left[E_{\alpha },T_{j}\left(t_{\alpha }\right)\right]}$$

where

(2) $$J\left[E_{\alpha },T_{i}\left(t_{\alpha }\right)\right]=\int_{t_{\alpha -∆\alpha }}^{t_{\alpha }}\exp\left[\frac{-E_{\alpha }}{RT_{i}\left(t\right)}\right]dt$$

and *p* is the number of the temperature programs, *T*(*t*). Statistical errors in *E_α_* were evaluated as described elsewhere [25]. 

The obtained isoconversional values of *E_α_* were then substituted into the equation of the compensation effect:

(3) $$\ln A_{\alpha }=a+bE_{\alpha }\;$$

to estimate the values of the pre-exponential factor. The *a* and *b* parameters of the compensation effect were determined by fitting the *lnA_i_* and *E_i_* pairs into Equation (3). The respective pairs were found by substituting the *f_i_*(*α*) reaction models into the linearized basic rate equation:

(4) $$\ln\left(\frac{d\alpha }{dt}\right)-\ln\left[f_{i}\left(\alpha \right)\right]=\ln A_{i}-\frac{E_{i}}{RT}$$

Each *lnA_i_* and *E_i_* pair was found from the intercept and slope of Equation (4) while plugging different *f_i_*(*α*) models in the left-hand side and plotting it against the reciprocal temperature. As recommended [26], four reaction models (the power law (P2, P3, P4) and Avrami–Erofeev (A2) were utilized in the compensation effect calculations.

The numerical values of the integral reaction model were estimated by inserting *E_α_* and *A_α_* into Equation (5).

(5) $$g\left(\alpha \right)=\sum_{\alpha }A_{\alpha }\;J\left[E_{\alpha },T_{i}\left(t_{\alpha }\right)\right]\;$$

The isoconversional activation energy for the solid-state polymerization under isothermal conditions was determined by the Friedman differential method [27] by plotting the left-hand side of Equation (6) against the reciprocal temperature.

(6) $$ln{\left(\frac{d\alpha }{dt}\right)}_{\alpha ,p}=ln\left[A_{\alpha }f\left(\alpha \right)\right]-\frac{E_{\alpha }}{RT_{\alpha ,p}}$$

## 4. Results and Discussion

**Figure 3:**
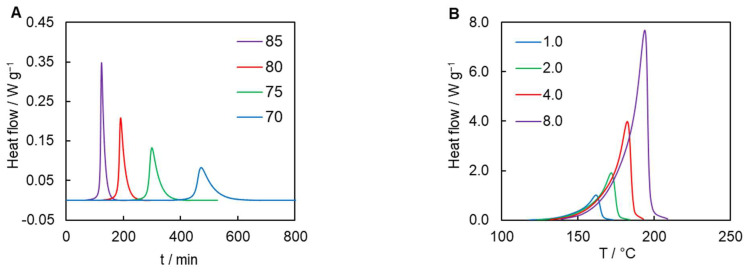
DSC curves for isothermal solid-state polymerization (numbers denote temperature in °C) (**A**), and nonisothermal liquid-state polymerization of the studied monomer (numbers denote heating rates in °C min^−1^) (**B**).

Polymerization of diacetylenes is a highly exothermic reaction due to the formation of the conjugated polymer chains (Figure 1). Therefore, the reaction progress is conveniently followed by DSC. In the kinetics studies by DSC, nonisothermal conditions are generally preferred because they avoid some technical issues as well as the diffusion limitations commonly encountered in the later stages of the process [28]. Naturally, this approach has been taken to measure the kinetics of liquid-state polymerization. However, the kinetics of the solid-state polymerization of diacetylenes has been routinely studied in isothermal conditions [13,14,15], including the present work. The reason is easy to understand by comparing the DSC data presented in Figure 3. The solid-state process has to be run below *T_m_* = 94 °C. As seen from nonisothermal DSC data, lowering the heating rate nearly 10 times lowers the peak temperature by less than 50 °C. To bring the peak temperatures under 94 °C, the heating rates would have to be lowered roughly below 0.01 °C min^−1^, which lies below the range typically accessible by DSC instruments because of the signal to noise problem. 

Figure 3A shows the DSC curves for solid-state polymerization measured in isothermal conditions. The reaction generates 128 ± 4 kJ mol^−1^ of heat, which closely matches previously reported values for the same monomer [13,16,21]. It must be noted that the solid-state polymerization clearly demonstrates distinct autocatalytic behavior. It is manifested by a well-defined induction period followed by acceleration. The duration of the induction period is seen to be up to about 6 h. Figure 3B presents temperature scans at different heating rates of the liquid-state polymerization of the monomer under study. As one can see, the reaction starts above 120 °C, i.e., roughly 30 °C higher than the melting temperature of the monomer. Remarkably, the liquid-state reaction produces almost three times more heat than the reaction in the crystalline phase, namely, 360 ± 5 kJ mol^−1^. Considering that the heat of melting of the monomer is 36.3 ± 0.8 kJ mol^−1^, such significant difference between the reaction heat values cannot be explained simply by the difference in the phase states of the reacting monomer. Rather, it means that the chemistry of the reaction in these phases is completely different. The net result of the solid-state reaction of diacetylenes proceeding with the formation of conjugated enyne structure (Figure 1) is the transformation of one π-bond into one σ-bond (per 1 monomer molecule). Apparently, the liquid-state reaction requires a larger number of breaking and forming bonds per monomer molecule. The liquid-state polymerization of the studied monomer can be proposed to occur as an intramolecular cyclization of diacetylenes, similar to the Hexadehydro Diels–Alder reaction [29]. In this case, two π-bonds are converted into two σ-bonds, which should produce a proportionally large amount of heat. 

**Figure 4:**
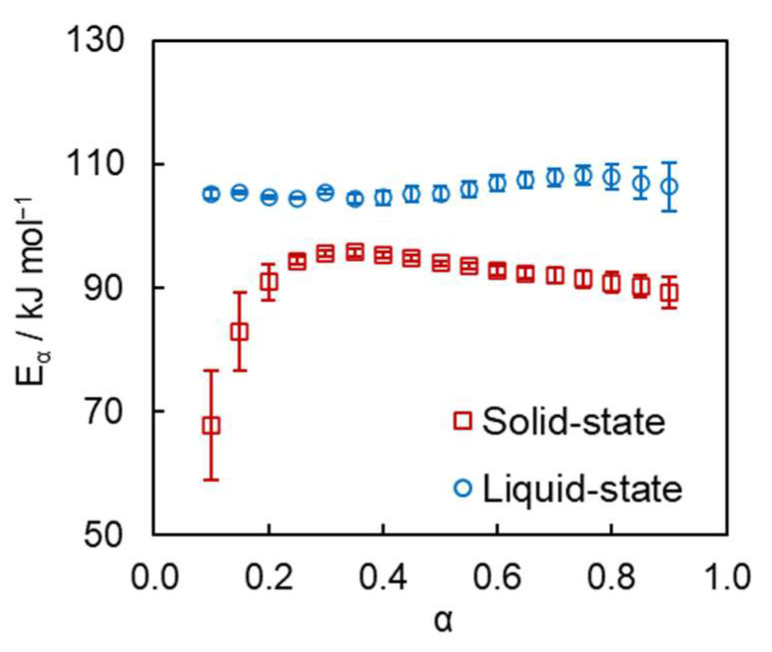
Isoconversional values of activation energy for solid- (squares) and liquid-state (circles) reactions.

The measured calorimetric data have been subjected to isoconversional kinetic analysis to quantify the reactivity of the monomer in the solid- and liquid-state reactions and to compare the kinetics for both processes. The calculated conversion dependencies of the activation energy *E_α_* for both reactions are presented in Figure 4. It is seen that the reactions demonstrate very different trends in the effective activation energy change with conversion. The effective activation energy of solid-state polymerization increases from 68 to 95 kJ mol^−1^, highlighting its complex kinetics that definitely comprises more than one rate-limiting step. It is interesting to mention that previous studies of the solid-state polymerization of 2,4-hexadiyne-1,6-diyl *bis*-(*p*-toluenesulfonate) by extraction [21], spectroscopic [17], and calorimetric techniques [16] report a single value of the activation energy equal to 94 ± 2 kJ mol^−1^. This means that the solid-state polymerization of the 2,4-hexadiyne-1,6-diyl *bis*-(*p*-toluenesulfonate) was treated as a single-step process, which obviously is not the case as demonstrated by our analysis. It should be noted that in all previous studies the activation energy was calculated from the temperature dependence of the reaction time related to a specific conversion (e.g., half-conversion time $t_{0.5}$) or the time related to the maximum polymerization rate ($t_{max}$, which is also roughly isoconversional). Such approaches produce inaccurate results because the respective equations are obtained by integration that assumes that the activation energy is independent of conversion and, thus, of time. This assumption holds true only for single-step processes. However, if the process rate is limited by more than one step, the resulting effective activation energy becomes conversion dependent [22,30], which invalidates the aforementioned assumption and introduces a systematic error in the resulting integrated equation for determining the activation energy. No such error occurs in the differential method used in the present work.

On the contrary, the liquid-state polymerization demonstrates a nearly constant effective activation energy in the range of *α* = 0.1–0.9, which signifies the existence of a well-defined rate-limiting step. The averaged value of the activation energy for this step is 106 ± 2 kJ mol^−1^. The natural logarithm of the pre-exponential factor *A_α_* determined via compensation effect (Equations (3) and (4)) is also nearly constant in the range of *α* = 0.1–0.9, and its average value equals 22.3 ± 0.4 (*A_α_* in s^−1^).

**Figure 5:**
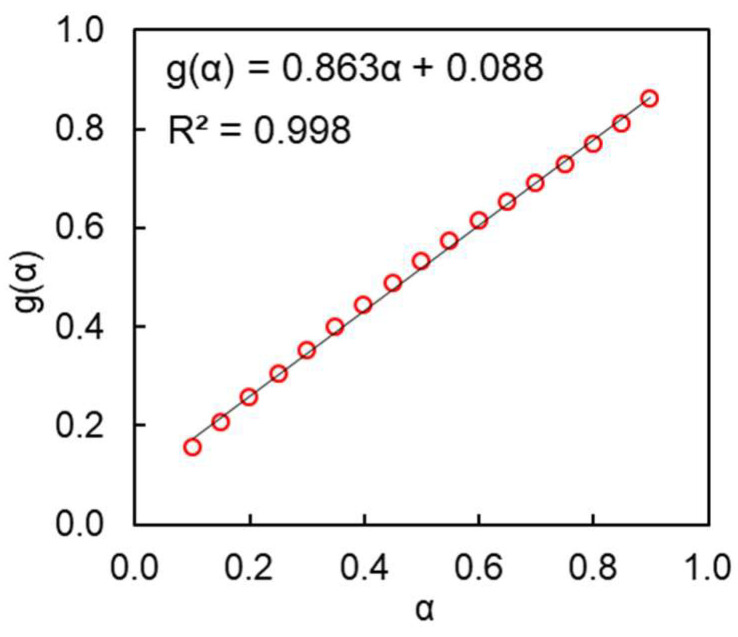
Experimental *g*(*α*) data for the studied reaction and their linear approximation.

It is also instructive to compare the reaction models for solid- and liquid-state polymerizations. As already mentioned, solid-state polymerization follows the reaction model of an autocatalytic type (Figure 3A). It is well known that the integral reaction models *g*(*α*) for the autocatalytic reactions (e.g., the Avrami–Erofeev models) present a sigmoid, i.e., distinctly nonlinear dependence on *α* [22]. Unfortunately, it is impossible to identify the exact form of *g*(*α*) for solid-state polymerization because it is not a single-step process. There is no such problem for the liquid-state reaction. For it, the experimental *g*(*α*) dependence is practically linear in a wide range of conversions (Figure 5). A linear dependence of *g*(*α*) on *α* means that the following holds true:

(7) $$g\left(\alpha \right)\equiv \int_{0}^{\alpha }\frac{d\alpha }{f\left(\alpha \right)}=B\alpha +C\;$$

where *B* and *C* are constants. Differentiation of the expression (7) with respect to *α* leads to:

(8) $$f\left(\alpha \right)=B^{-1}\equiv B^{-1}{\left(1-\alpha \right)}^{0}$$

Thus, a linear *g*(*α*) on *α* plot corresponds to a zero reaction-order dependence on (1 − *α*) [31]. The reaction rate profile of a zero-order reaction traces the temperature dependence of the reaction rate constant *k*(*T*), which means that under the constant heating rate conditions the rate rises exponentially with increasing temperature until the reaction completes. Since the DSC signal is directly proportional to the reaction rate, the respective reaction heat flow should be expected to rise exponentially and drop quickly once the reaction reaches completion. This behavior should result in generating asymmetric DSC peaks such as those measured experimentally for the reaction under study (Figure 3B).

**Figure 6:**
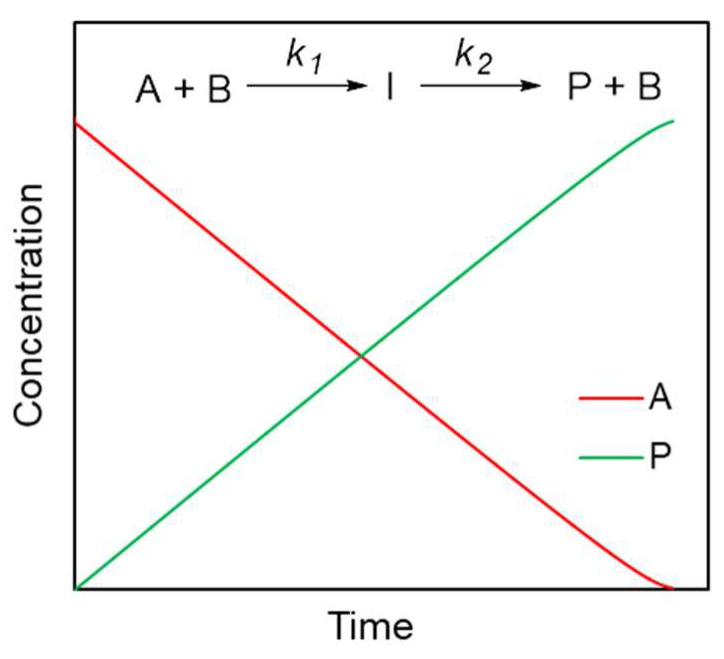
Simulation of concentration profiles for catalytic transformation of reactant A through two consecutive reactions with $k_{1}/k_{2}=100$ and ${C_{0}(A)/C}_{0}(B)=100$. The results obtained with the online kinetics simulator [33].

A simple reaction scheme that can explain the zero-order kinetics is as follows. Let us consider a two-step process that involves the formation of intermediate I via a reaction of reactant A with catalyst B followed by conversion of I into product P (Figure 6). When the concentration of B is much lower than that of A, and *k*_1_ >> *k*_2_, then the product of A and B concentrations as well as the concentration of I becomes practically constant in a wide range of times. In this case, the rates of the corresponding reactions also remain nearly constant, giving rise to a linear dependence of the concentrations of A and P on time (Figure 6), which constitutes the zero-order kinetics. The heat flow generated by such a process of conversion A to P will also give rise to the zero-order reaction rate law. Although presently the mechanism of the liquid-state polymerization of diacetylenes is virtually unknown, it is not unreasonable to expect that this two-step reaction model is applicable if the polymerization is catalyzed by some impurities, e.g., trace amounts of copper-based catalyst used in diol synthesis or water. The presence of the latter one is detected by ^1^H NMR spectroscopy in the initial monomer sample (Figure S1). Such catalysis is not uncommon, for example, in the cyanate esters polymerization that is known to be catalyzed by residual phenols or traces of water [32]. 

Important information about the rate-limiting step of the studied reaction can also be obtained from the analysis of its activation entropy ($\Delta S^{\neq }$) [34]. According to the activated complex theory [35] for a reaction, proceeding in a solution $\Delta S^{\neq }$ can be calculated as

(9) $$\Delta S^{\neq }=R\left[\ln\left(\frac{A_{i}h}{k_{B}T}\right)-1\right]\;$$

where h and $k_{B}$ represent, respectively, the Planck and Boltzmann constants, and A_i_ is an intrinsic value of the pre-exponential factor. Considering the melt of the reacting monomer as a solution in itself, we can apply Equation (9) to the studied reaction. Since the intrinsic value of the pre-exponential factor is unknown, we have to use the effective one reported earlier. This yields the $\Delta S^{\neq }$ value of −78 ± 3 J K^−1^ mol^−1^. One should be aware that the effective nature of the pre-exponential factor propagates into the calculated activation entropy as well [34]. Nevertheless, it is important to notice that the activation entropy has a sufficiently large negative value. It means that a transition state formed in the rate-limiting step is more ordered than the reactants, from which it is formed. This is typically found in bimolecular reactions [36]. It can also be expected in other reactions, whose rate-limiting step has an associative nature. It is worth noting that we have determined negative values of the activation entropy for the reactions of polymerization [37] and 1,3-dipolar cycloaddition [38]. 

The reaction product of liquid-state polymerization is a black brittle material insoluble in usual organic solvents. Its aqueous dispersion is strongly acidic, apparently, due to the presence of p-toluenesulfonic acid formed during the reaction. The presence of the p-toluenesulfonic acid among the products of the liquid-state reaction is also confirmed by FTIR (Figures S3–S5). The initial monomer features an absorption band at 1360 cm^−1^ that corresponds to the stretching vibration of the S=O bond in its sulfonate group. Upon polymerization, this band transforms into the 1175 cm^−1^ absorption band corresponding to the hydrated sulfonic acid group of p-toluenesulfonic acid [39]. Complete disappearance of the 1360 cm^−1^ absorption band in the liquid-state polymerization product signifies the quantitative nature of elimination of the p-toluenesulfonate group. Most likely, this liquid-state reaction is initiated by residual water detected in the initial monomer sample (Figure S1). It is remarkable that despite the fact that residual water is also present in solid-state polymerization, this process does not involve elimination of the p-toluenesulfonate group as evidenced by the presence of absorption at 1360 cm^−1^ and the absence of the 1175 cm^−1^ absorption band in the resulting polymerization product. This observation is consistent with the fact that in the solid state the reaction proceeds via 1,4-addition without involving the side-groups [16]. The 1175 cm^−1^ absorption band also does not appear after subjecting this product to the same nonisothermal heating schedule as the one used for liquid-state polymerization. Evidently, the exclusive formation of p-toluenesulfonic acid during liquid-state polymerization is caused by the mechanistic features of polymerization rather than thermal instability of the monomer. X-ray powder diffraction analysis reveals the semicrystalline nature of the liquid-state reaction product, whereas solid-state polymerization results in a highly crystalline polymer (Figures S6 and S7). It is important to note that a part of the XRD patterns of the liquid-state reaction product coincides with those of crystalline p-toluenesulfonic acid hydrate (Figure S7). The acid is highly hygroscopic, so it readily forms a hydrate upon exposure to the atmospheric humidity. The presence of the acid in combination with the less than 2.5 % mass loss during polymerization (Figure S8) indicates that nearly all p-toluenesulfonic acid remains entrapped in the polymerization product.

**Figure 7:**
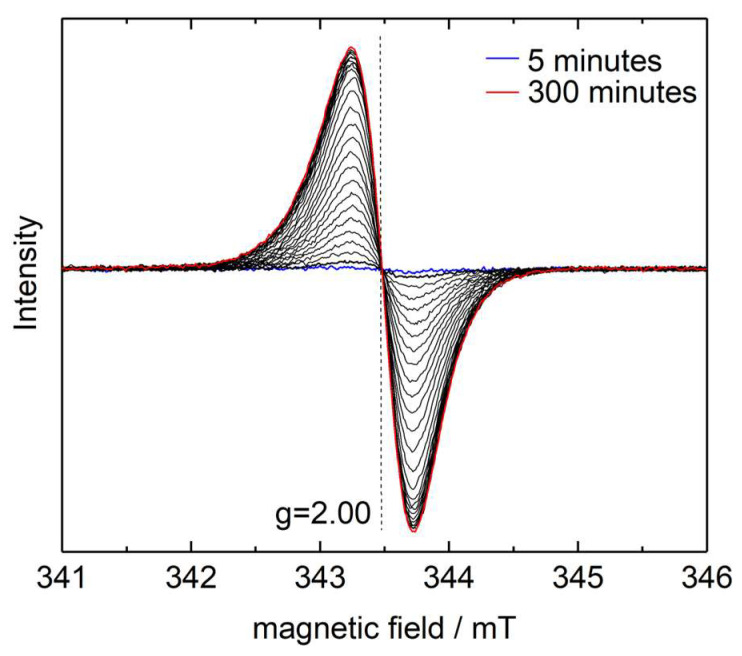
Evolution of the EPR spectra during measurement at 130 °C. Measurement interval is ~15 min.

A proposed mechanism of the solid-state polymerization of diacetylenes includes the formation of certain reactive radical species [12,40]. Therefore, to obtain further insights into the liquid-state reaction mechanism, we have applied in situ EPR spectroscopy. Although, the lifetime of the respective radicals is too short to be detected even at ambient temperature. For example, the half-life of cumulene-type biradicals initially formed in the solid-state reaction does not exceed 5 h at −183 °C) [12]. Of course, it is unrealistic to detect such radicals by in situ EPR in the liquid-state polymerization of diacetylenes that occurs on the scale of hours above 120 °C. However, we have assumed that such reactive species may participate in the radical-transfer processes with the formation of more stable secondary radical centers. Indeed, a single absorption line with a g-factor of 2.00 and a width of 5 Gauss appears in the EPR spectra (Figure 7) during isothermal measurements in the temperature range of 130–145 °C, which corresponds to the initial stages of the liquid-state polymerization (see Figure 3). The intensity of the detected EPR line increases with time. Also, the radical generation rate increases with increasing temperature. It should be noted that no signals were observed in the EPR spectra of the initial monomer. Upon cooling, the intensity of the absorption line of the reaction mixture remains nearly the same even after a few days of storage at room temperature. It means that the radicals formed are stable and the process of their formation is irreversible. Their concentration in the reaction mixture has been estimated to be around (6.67 ± 0.05) × 10^18^ spin g^−1^. This suggests that roughly one of two hundred monomeric units in the polymer bears a radical center. The radical centers, apparently, are embedded into the polymer matrix because their concentration remains almost the same after extraction of low molecular product by tert-butanol.

**Figure 8:**
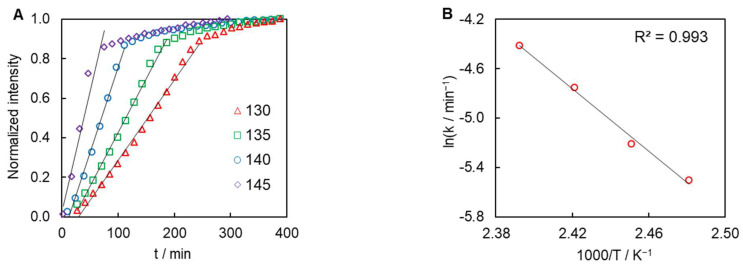
Normalized intensity of EPR signal vs. time at different temperatures (**A**) and temperature dependence of the generation rate constant (**B**).

Remarkably, the intensity of the EPR signal measured at different temperatures increases linearly with time up to 0.85 of relative intensity (Figure 8A). This means that just as the heat release rate does, the rate of generation of free radicals follows the zero-order kinetics (Equations (10) and (11)).

(10) $$\frac{dI}{dt}=k(T)\;$$

(11) I=k(T)t 

The temperature dependence of the generation rate constant *k*(*T*) calculated from the linear part of the data presented in Figure 8A follows the Arrhenius equation (Figure 8B) and gives rise to the activation energy of 105 ± 6 kJ mol^−1^. The corresponding value of the natural logarithm of the pre-exponential factor *A* is 25.6 ± 1.9 (*A* in min^−1^) or 21.5 ± 1.9 (*A* in s^−1^).

Clearly, the polymerization kinetics determined from the heat release data as measured by DSC and from the free radical generation process as measured by EPR are strikingly similar. The activation energy is 106 ± 2 vs. 105 ± 6 kJ mol^−1^ and the natural logarithmic value of the pre-exponential factor is 22.3 ± 0.4 vs. 21.5 ± 1.9 (*A_α_* in s^−1^). In addition, both kinetics follow the zero-order reaction model. Apparently, either the radical generation process limits the overall rate of polymerization and, thus, the heat release rate or both processes have the same rate-limiting step.

## 5. Conclusions

For the first time, a detailed investigation of the liquid-state polymerization of diacetylenes has been performed. The thermally initiated liquid-state polymerization of 2,4-hexadiyne-1,6-diyl *bis*-(*p*-toluenesulfonate) demonstrates the thermodynamic and kinetic parameters that differ significantly from those for the solid-state reaction. In particular, the reaction heat values for liquid- and solid-state reactions differ by 2.8 times (360 ± 5 vs. 128 ± 4 kJ mol^−1^). Isoconversional kinetic analysis applied to the calorimetric data reveals that liquid-state polymerization occurs via a well-defined rate-limiting step characterized by the effective activation energy of 106 ± 2 kJ mol^−1^, whereas the effective activation energy of the solid-state process varies in a wide range, suggesting that the corresponding rate is determined by more than one step. Also, the liquid-state polymerization follows a rather unusual zero-order reaction model, whereas the solid-state reaction demonstrates autocatalytic behavior. The liquid-state reaction results in the quantitative elimination of the *p*-toluenesulfonate group and the formation of *p*-toluenesulfonic acid and a polymeric product with stable free radical centers. The similarity of the kinetic parameters derived from the DSC and in situ EPR data, as well as the adherence of the respective kinetics to the same zero-order reaction model, apparently, indicates that the overall rate of polymerization is either limited by the radical generation or both processes have the same rate-limiting step. Further studies are needed to see whether the discovered striking differences between liquid- and solid-state polymerization are characteristic for other diacetylene monomers.

## Data Availability

The data presented in this study are available on request from the corresponding authors.

## Supplementary Materials

The following supporting information can be downloaded at: https://www.mdpi.com/article/10.3390/polym16010007/s1.
