# Supplementary material for: Kinetics and Mechanism of Liquid-State Polymerization of 2,4-Hexadiyne-1,6-diyl bis-(p-toluenesulfonate) as Studied by Thermal Analysis
Source: Polymers (Basel). 2023 Dec 19;16(1):7. doi: 10.3390/polym16010007 (PMC10780683; doi:10.3390/polym16010007)
Supplement: Supplementary file 1 [file polymers-16-00007-s001.zip › polymers-2724731-supplementary.pdf]

Kinetics and Mechanism of Liquid-State Polymerization of 2,4-Hexadiyne-1,6-diyl *bis*-(*p*-toluenesulfonate) as Studied by Thermal Analysis

Andrey Galukhin [1,*], Alexander Kachmarzhik [1], Alexander Rodionov [2], Georgy Mamin [2], Marat Gafurov [2] and Sergey Vyazovkin [3,*]

1. Alexander Butlerov Institute of Chemistry, Kazan Federal University, 18 Kremlevskaya Street, 420008 Kazan, Russia; sasha.kachma@mail.ru
2. Institute of Physics, Kazan Federal University, 18 Kremlevskaya Street, 420008 Kazan, Russia; rodionovshurik@yandex.ru (A.R.); georgemamin@gmail.com (G.M.); mgafurov@gmail.com (M.G.)
3. Department of Chemistry, University of Alabama at Birmingham, 901 S. 14th Street, Birmingham, AL 35294, USA

* Correspondence: and_galuhin@mail.ru (A.G.); vyazovkin@uab.edu (S.V.)

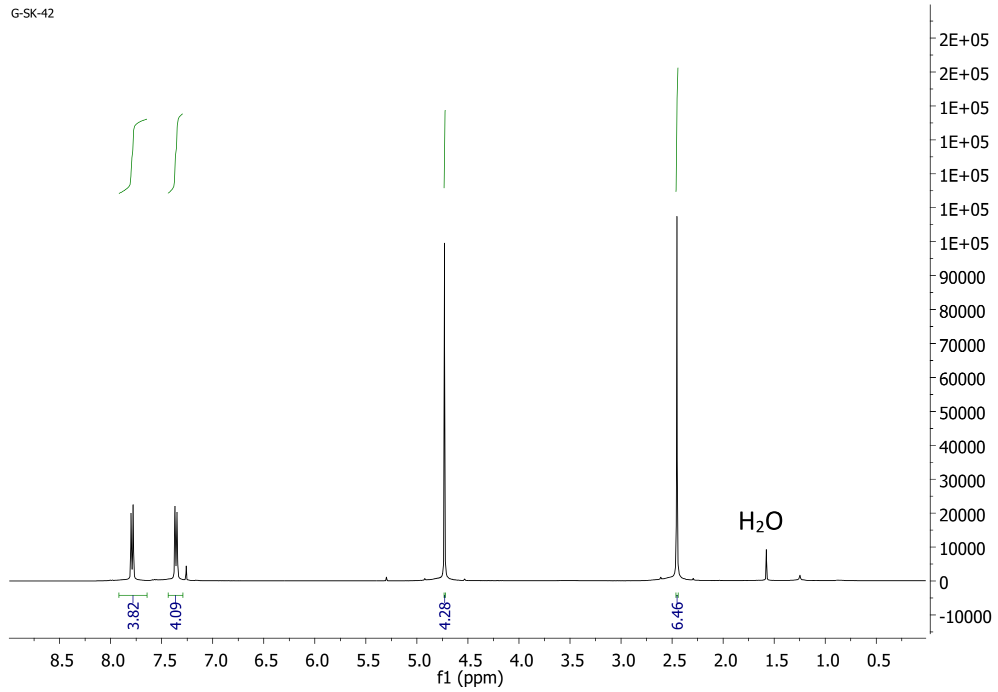

Figure S1. ^{1}H NMR spectrum of the 2,4-hexadiyne-1,6-diyl bis-(p-toluenesulfonate).

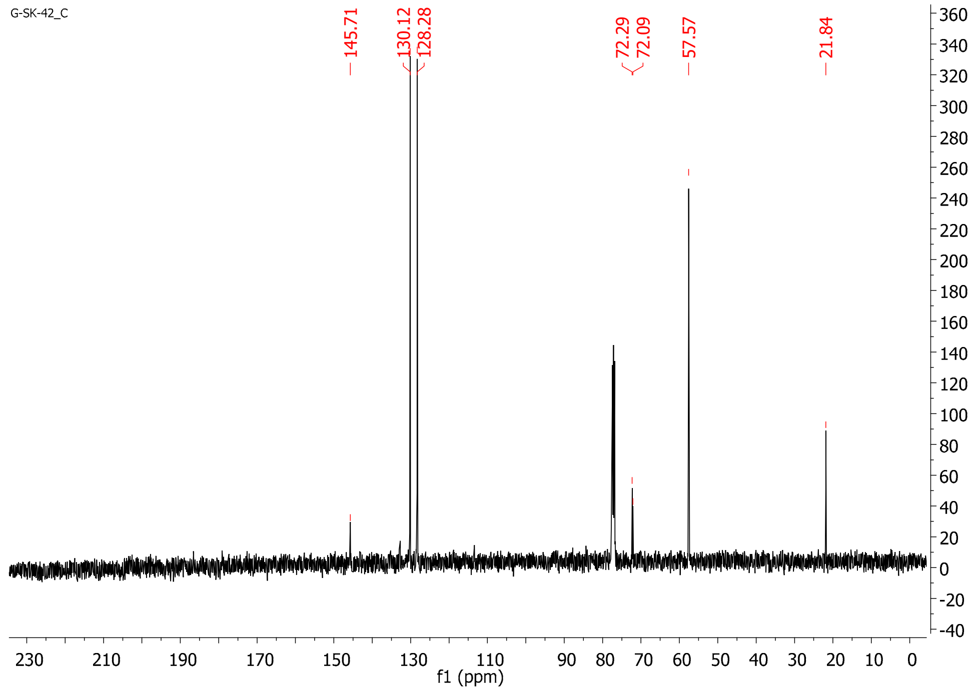

Figure S2. ^{13}C NMR spectrum of the 2,4-hexadiyne-1,6-diyl bis-(p-toluenesulfonate).

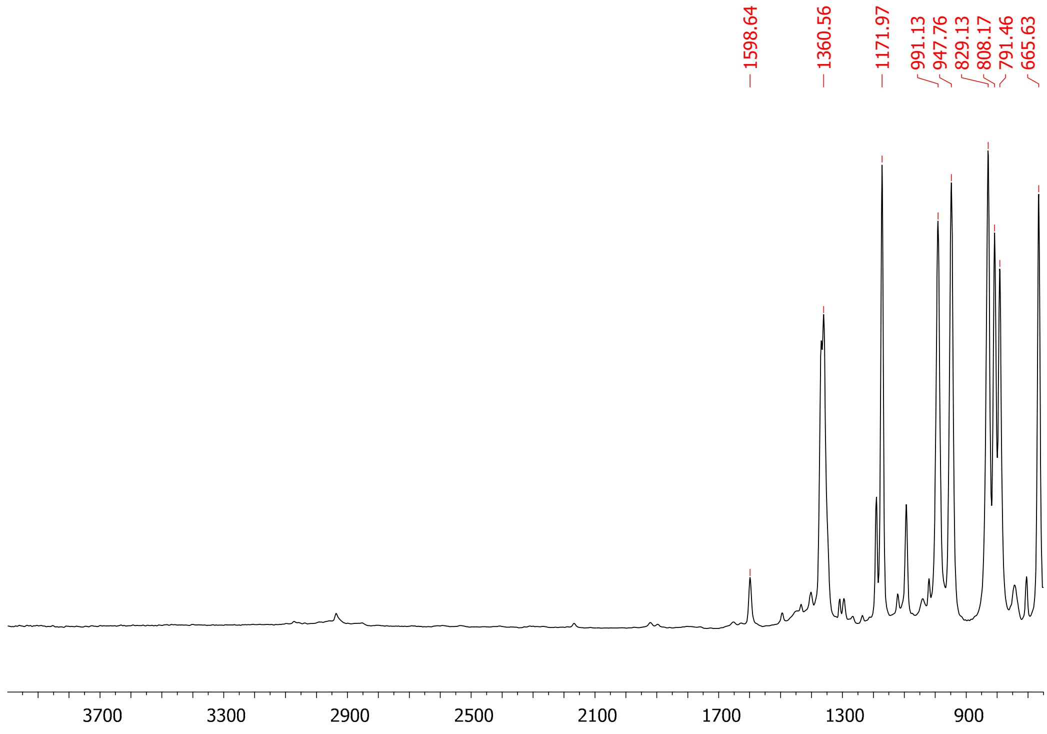

Figure S3. FTIR spectrum of 2,4-hexadiyne-1,6-diyl bis-(p-toluenesulfonate).

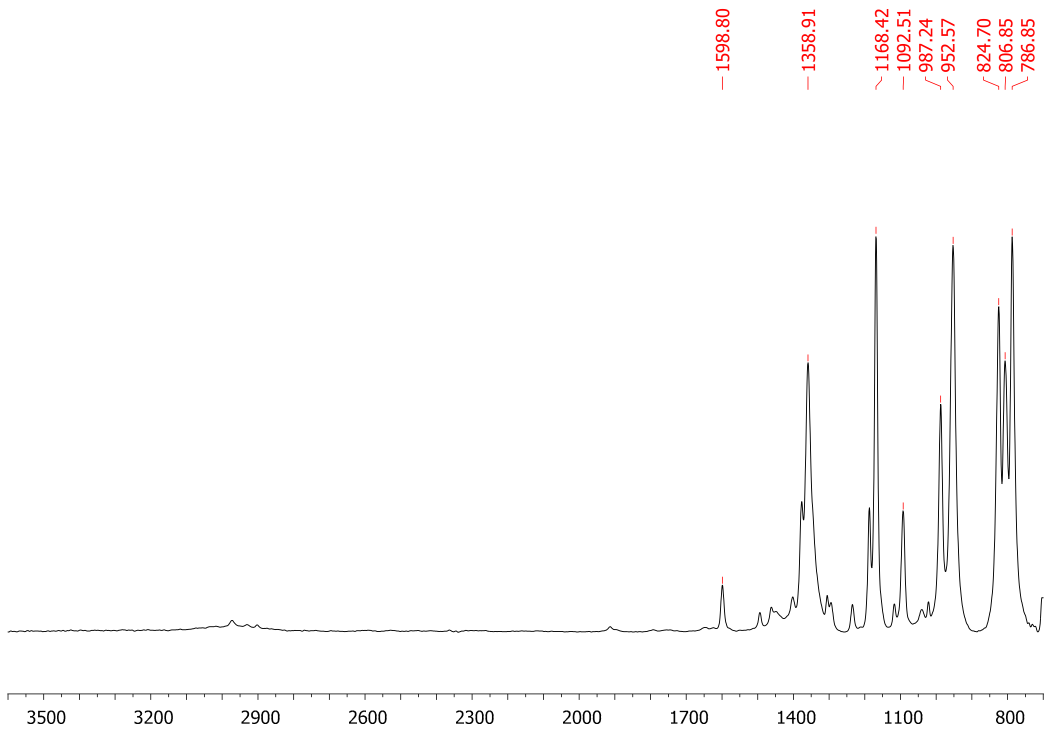

Figure S4. FTIR spectrum of the solid-state reaction product.

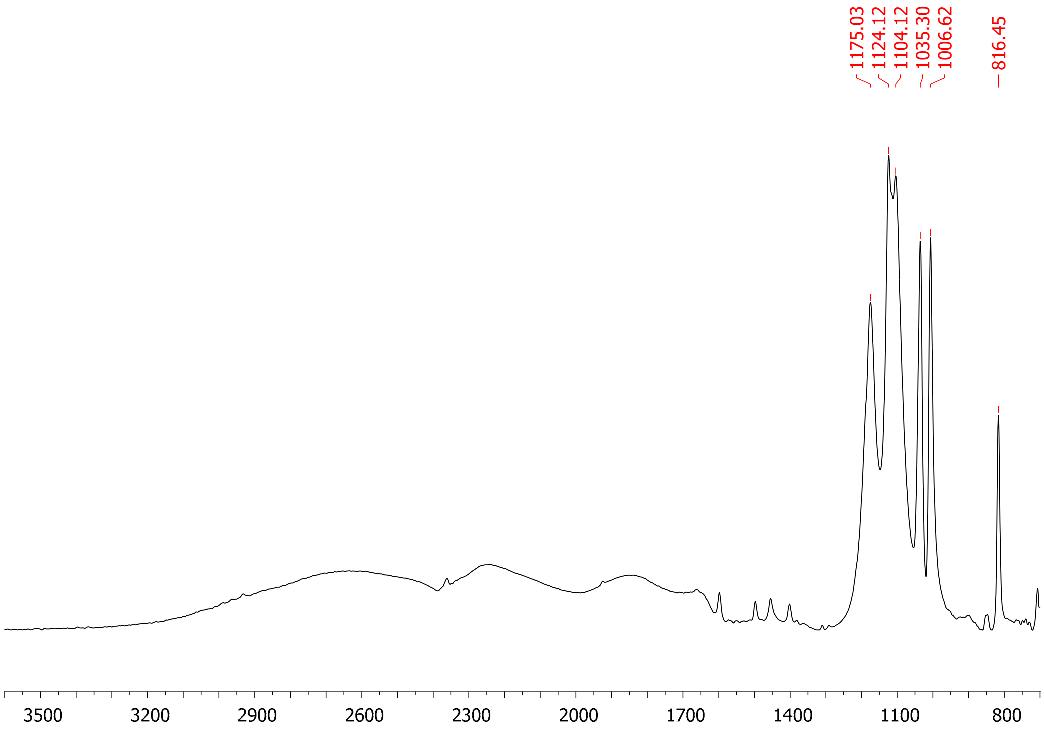

Figure S5. FTIR spectrum of the liquid-state reaction product.

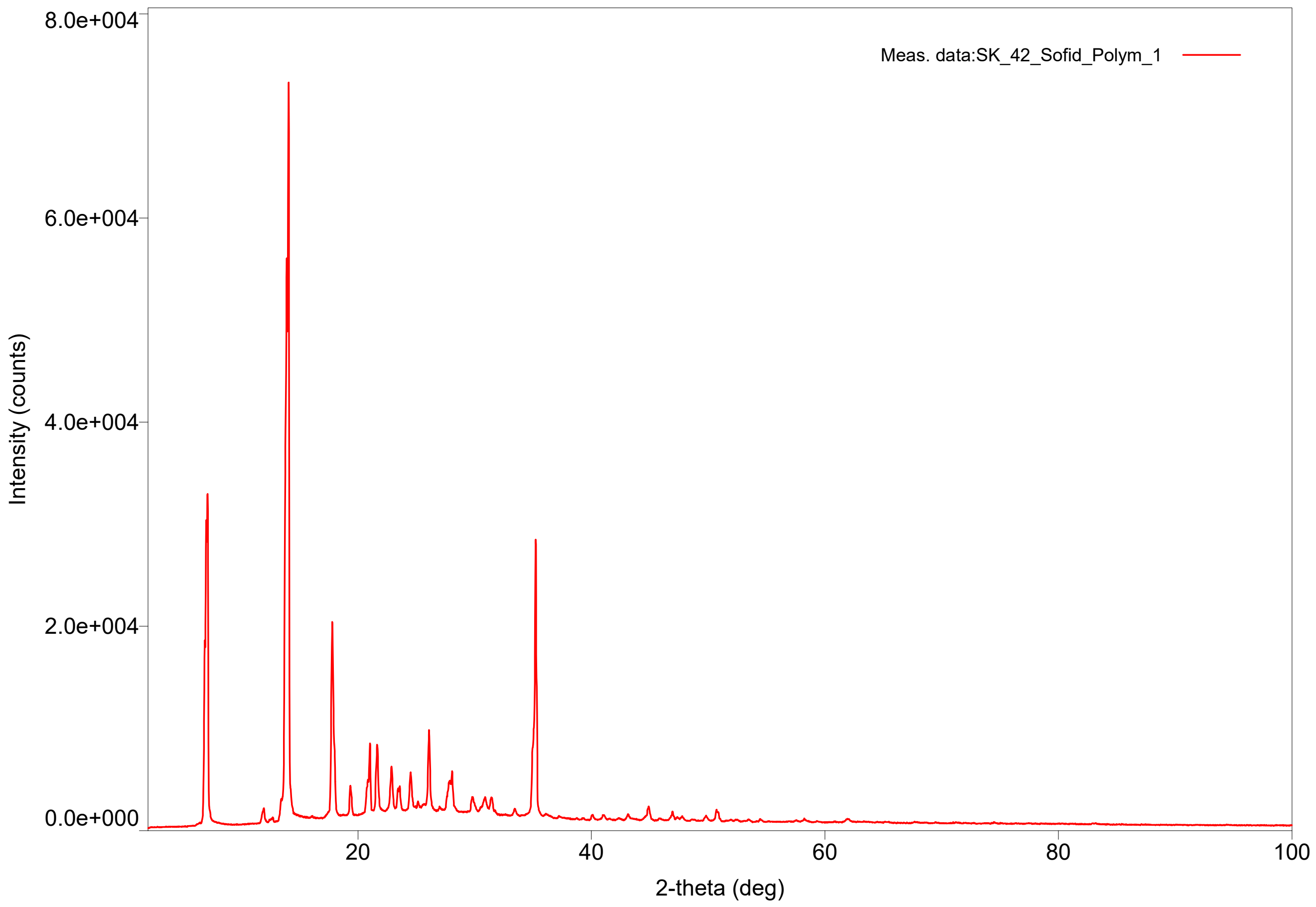

Figure S6. X-ray powder diffraction patterns of the solid-state reaction product.

Table S1. Parameters of the XRD patterns for the solid-state reaction product.

No.	2-theta(deg)	d(ang.)	Height(counts)	FWHM(deg)	Int. I(counts deg)	Int. W(deg)	Asym. factor
1	6.880(4)	12.837(8)	11293(106)	0.22(6)	3068(729)	0.27(7)	0.5(2)
2	6.986(10)	12.642(17)	9447(97)	0.12(6)	1412(1191)	0.15(13)	0.35(14)
3	7.144(3)	12.364(6)	17542(132)	0.12(3)	2496(1016)	0.14(6)	3.1(8)
4	11.936(6)	7.409(4)	943(31)	0.232(6)	259(5)	0.274(14)	2.7(4)
5	12.706(5)	6.961(3)	269(16)	0.285(15)	87(4)	0.32(3)	5.0(15)
6	13.451(5)	6.577(3)	1465(38)	0.80(2)	1381(58)	0.94(6)	0.202(19)
7	13.771(4)	6.4251(18)	22835(151)	0.138(4)	3552(199)	0.156(10)	1.2(3)
8	13.8853(16)	6.3726(7)	32894(181)	0.144(8)	5453(378)	0.166(12)	0.58(8)
9	14.0678(9)	6.2903(4)	46113(215)	0.101(4)	5165(305)	0.112(7)	2.52(12)
10	17.692(4)	5.0090(12)	3766(61)	0.075(11)	347(195)	0.09(5)	1.5(6)
11	17.783(4)	4.9838(11)	12932(114)	0.155(19)	2464(307)	0.19(3)	0.7(2)
12	17.993(5)	4.9259(14)	3585(60)	0.15(4)	669(194)	0.19(6)	1.8(8)
13	19.323(3)	4.5899(8)	1990(45)	0.176(4)	407(7)	0.205(8)	0.45(4)
14	20.714(11)	4.285(2)	1258(35)	0.15(2)	236(24)	0.19(2)	0.7(2)
15	20.849(12)	4.257(2)	1743(42)	0.183(14)	394(269)	0.23(16)	0.61(18)
16	21.0296(15)	4.2210(3)	4738(69)	0.096(3)	558(23)	0.118(6)	2.19(16)
17	21.541(5)	4.1220(10)	2722(52)	0.11(2)	403(112)	0.15(4)	0.8(3)
18	21.640(4)	4.1032(8)	4196(65)	0.132(6)	735(113)	0.18(3)	0.57(11)
19	22.873(4)	3.8848(7)	2877(54)	0.217(3)	785(8)	0.273(8)	1.55(13)
20	23.391(4)	3.8000(7)	1419(38)	0.210(19)	327(20)	0.23(2)	0.34(10)
21	23.582(10)	3.7697(15)	1062(33)	0.082(14)	98(23)	0.09(2)	1.0(4)
22	24.437(10)	3.6396(14)	1898(44)	0.16(3)	320(61)	0.17(4)	0.8(2)
23	24.501(9)	3.6303(13)	1298(36)	0.172(16)	237(62)	0.18(5)	0.20(8)
24	25.13(2)	3.541(3)	426(21)	0.12(3)	69(8)	0.16(3)	0.9(8)
25	26.079(4)	3.4141(5)	5012(71)	0.157(3)	836(26)	0.167(8)	1.33(12)
26	26.190(16)	3.400(2)	426(21)	0.74(5)	337(20)	0.79(9)	4.8(13)
27	27.799(10)	3.2066(12)	1853(43)	0.441(8)	869(15)	0.469(19)	0.65(6)
28	28.075(5)	3.1758(5)	1833(43)	0.096(6)	188(13)	0.103(9)	2.3(5)
29	29.730(8)	3.0026(8)	1033(32)	0.300(9)	379(15)	0.37(3)	0.26(4)
30	30.891(10)	2.8924(9)	963(31)	0.425(12)	495(10)	0.51(3)	2.0(2)
31	31.446(9)	2.8425(8)	1162(34)	0.246(9)	347(12)	0.298(19)	1.9(4)
32	31.741(16)	2.8169(14)	274(17)	0.11(3)	35(7)	0.13(3)	1.9(13)
33	33.419(13)	2.6791(10)	502(22)	0.202(17)	134(6)	0.27(2)	1.1(4)
34	34.939(4)	2.5659(3)	4443(67)	0.308(9)	1680(37)	0.378(14)	0.27(2)
35	35.2302(6)	2.54542(4)	20853(144)	0.1058(10)	2887(46)	0.138(3)	2.04(5)
36	36.08(3)	2.487(2)	188(14)	0.42(4)	98(7)	0.52(8)	0.39(14)
37	37.184(11)	2.4160(7)	169(13)	0.21(3)	68(4)	0.40(5)	0.32(9)
38	40.063(18)	2.2488(10)	376(19)	0.154(17)	63(5)	0.17(2)	0.9(4)

39	41.02(2)	2.1984(12)	341(18)	0.23(2)	96(6)	0.28(3)	1.2(6)
40	43.11(3)	2.0968(14)	397(20)	0.21(4)	125(8)	0.31(4)	1.0(6)
41	44.850(8)	2.0193(3)	994(32)	0.208(12)	295(8)	0.297(17)	0.76(14)
42	45.787(19)	1.9801(8)	160(13)	0.15(3)	29(4)	0.18(4)	0.4(3)
43	46.969(6)	1.9330(2)	578(24)	0.209(8)	141(4)	0.245(16)	4.8(10)
44	47.368(10)	1.9176(4)	249(16)	0.159(17)	47(3)	0.19(3)	3.2(12)
45	47.794(10)	1.9015(4)	300(17)	0.218(17)	77(4)	0.26(3)	4.2(12)
46	48.57(3)	1.8729(13)	86(9)	0.24(4)	22(4)	0.25(7)	0.3(2)
47	49.809(13)	1.8292(5)	363(19)	0.226(13)	95(4)	0.26(2)	1.7(4)
48	50.690(7)	1.7995(2)	886(30)	0.207(7)	229(6)	0.258(16)	0.36(7)
49	51.97(3)	1.7582(8)	112(11)	0.19(4)	33(5)	0.30(7)	2.8(18)
50	52.35(2)	1.7462(7)	137(12)	0.19(3)	33(3)	0.24(5)	0.4(2)
51	53.458(10)	1.7126(3)	163(13)	0.16(3)	31(4)	0.19(4)	1.4(9)
52	54.461(13)	1.6835(4)	236(15)	0.132(13)	33(4)	0.14(3)	1.1(5)
53	57.416(13)	1.6036(3)	129(11)	0.17(4)	36(5)	0.28(6)	0.20(10)
54	58.221(13)	1.5833(3)	237(15)	0.19(3)	78(5)	0.33(4)	0.8(2)
55	61.82(3)	1.4995(8)	238(15)	0.40(6)	160(7)	0.67(7)	0.37(18)
56	67.71(2)	1.3827(4)	95(10)	0.13(3)	17(3)	0.18(5)	1.4(14)
57	82.907(9)	1.16357(10)	86(9)	0.31(3)	30(3)	0.34(7)	0.20(12)

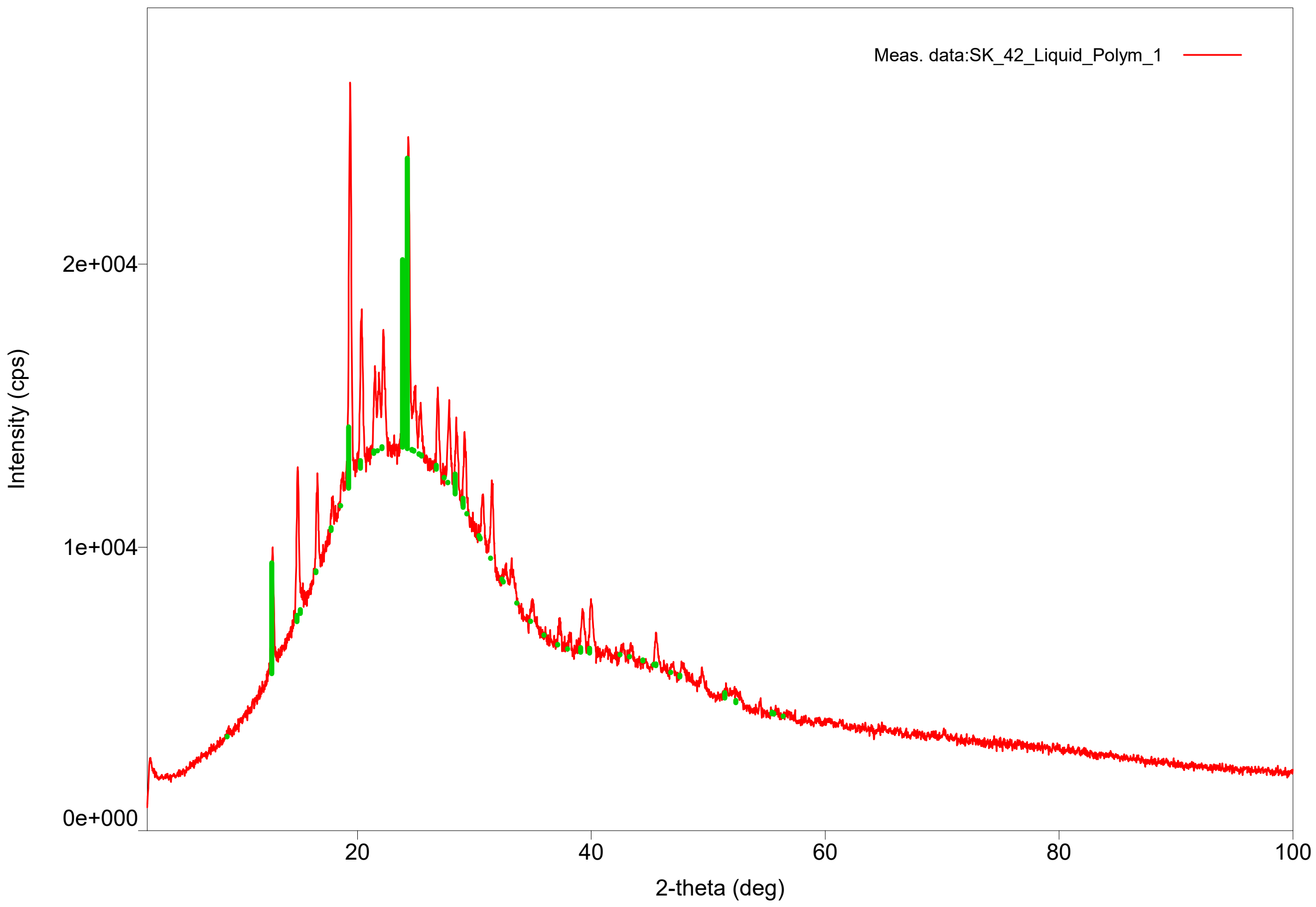

Figure S7. X-ray powder diffraction patterns of the liquid-state reaction product. Green patterns correspond to *p*-toluenesulfonic acid hydrate (DB card number 00-054-2253).

Table S2. Parameters of the XRD patterns for the liquid-state reaction product.

No.	2-theta(deg)	d(ang.)	Height(counts)	FWHM(deg)	Int. I(counts deg)	Int. W(deg)	Asym. factor
1	12.731(6)	6.948(4)	705(27)	0.222(6)	207(4)	0.294(17)	1.14(15)
2	14.885(7)	5.947(3)	903(30)	0.214(7)	269(6)	0.298(16)	1.10(17)
3	16.545(11)	5.354(4)	502(22)	0.200(9)	107(6)	0.21(2)	0.9(2)
4	17.84(3)	4.968(9)	158(13)	0.18(3)	30(6)	0.19(5)	1.1(2)
5	18.588(10)	4.770(3)	126(11)	0.21(4)	33(5)	0.26(6)	0.2(2)
6	19.359(3)	4.5813(8)	2335(48)	0.215(3)	614(7)	0.263(9)	1.07(7)
7	20.323(9)	4.3661(19)	875(30)	0.240(8)	241(8)	0.275(19)	0.88(14)
8	21.454(8)	4.1385(15)	363(19)	0.69(4)	267(12)	0.74(7)	0.20(5)
9	22.202(4)	4.0007(7)	545(23)	0.217(11)	128(8)	0.23(3)	0.64(15)
10	23.906(10)	3.7192(15)	725(27)	0.254(12)	196(10)	0.27(2)	0.77(13)
11	24.330(5)	3.6553(7)	1758(42)	0.239(5)	448(17)	0.255(16)	0.99(8)
12	24.92(2)	3.570(3)	333(18)	0.39(4)	139(15)	0.42(7)	2.2(7)
13	25.37(2)	3.508(3)	273(17)	0.27(2)	77(5)	0.28(4)	1.0(3)
14	26.841(14)	3.3188(17)	463(22)	0.212(10)	106(6)	0.23(2)	0.7(2)
15	27.806(13)	3.2058(15)	431(21)	0.259(13)	129(5)	0.30(3)	0.77(16)
16	28.449(14)	3.1348(15)	402(20)	0.254(13)	118(4)	0.29(3)	0.84(18)
17	29.146(13)	3.0615(13)	433(21)	0.241(11)	120(4)	0.28(2)	0.75(16)
18	30.71(3)	2.909(2)	276(17)	0.24(2)	80(6)	0.29(4)	1.5(8)
19	31.49(2)	2.8391(17)	452(21)	0.227(15)	118(8)	0.26(3)	0.9(3)
20	33.17(2)	2.6985(17)	144(12)	0.45(6)	97(9)	0.67(12)	0.7(4)
21	34.99(4)	2.562(3)	130(11)	0.24(6)	42(6)	0.32(8)	2(2)
22	37.28(5)	2.410(3)	156(12)	0.21(5)	40(7)	0.25(6)	1.6(18)
23	39.26(2)	2.2927(13)	238(15)	0.30(3)	92(5)	0.38(5)	1.5(4)
24	39.956(6)	2.2546(3)	316(18)	0.299(19)	115(5)	0.37(4)	0.8(2)
25	45.55(2)	1.9899(10)	207(14)	0.21(3)	54(4)	0.26(4)	1.7(9)
26	47.69(6)	1.905(2)	87(9)	0.34(11)	48(5)	0.55(12)	0.2(3)
27	49.50(2)	1.8398(8)	111(11)	0.21(3)	31(3)	0.28(6)	3.2(11)
28	52.09(12)	1.754(4)	57(8)	1.18(10)	72(9)	1.3(3)	1.1(5)

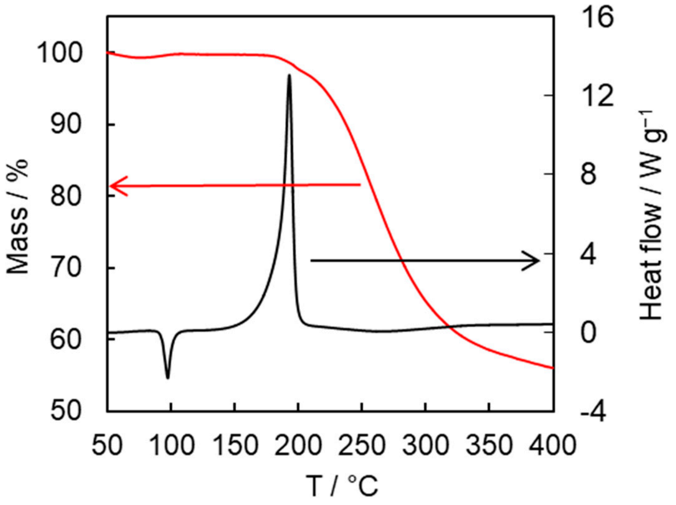

Figure S8. TG and DSC curves for liquid-state polymerization of the studied monomer measured at 10 °C min^{-1}.
